# Supplementary material for: Association between Uremic Toxin Concentrations and Bone Mineral Density after Kidney Transplantation
Source: Toxins (Basel). 2020 Nov 13;12(11):715. doi: 10.3390/toxins12110715 (PMC7696468; doi:10.3390/toxins12110715)
Supplement: Supplementary file 1 [file toxins-12-00715-s001.pdf]

Supplementary Materials: Association between Uremic Toxin Concentrations and Bone Mineral Density after Kidney Transplantation

Benjamin Batteux Sandra Bodeau, Camille André, Anne-Sophie Hurtel-Lemaire, Valérie Gras-Champel, Isabelle Desailly-Henry, Kamel Masmoudi, Youssef Bennis, Ziad A. Massy, Saïd Kamel, Gabriel Choukroun and Sophie Liabeuf

Table S1. Statistical significance (p value) of Spearman's correlation coefficients for UT concentrations upon transplantation.

UT Concentrations	pCS	CMPF	IxS	pCG	HA	TMAO	IAA
**pCS**		0.0530	0.0330	<0.0001	0.0005	0.0133	<0.0001
**CMPF**	0.0530		0.0017	0.0433	0.0009	0.0016	0.0102
**IxS**	0.0330	0.0017		<0.0001	<0.0001	<0.0001	0.0338
**pCG**	<0.0001	0.0433	<0.0001			<0.0001	<0.0001
**HA**	0.0005	0.0009	<0.0001	<0.0001		<0.0001	<0.0001
**TMAO**	0.0133	0.0016	<0.0001	<0.0001	<0.0001		<0.0001
**IAA**	<0.0001	0.0102	0.0338	<0.0001	<0.0001	<0.0001	

CMPF, 3-carboxy-4-methyl-5-propyl-furanpropionic acid; HA, hippuric acid; IAA, indole-3-acetic acid; IxS, indoxylsulfate; pCG, p-cresylglucuronide; pCS, p-cresylsulfate; TMAO, trimethylamine-N-oxide; UT, uremic toxin.

Table S2. Changes in BMD at M12 and M24, by subgroup.

**Time after Transplantation**	**Lumbar Spine**			**Femoral Neck**			**Total Hip**		
	**BMD Change (g/cm²), *m ± SD***	**BMD Change (%), *m ± SD***	***p* value**	**BMD Change (g/cm²), *m ± SD***	**BMD Change (%), *m ± SD***	***p* value**	**BMD Change (g/cm²), *m ± SD***	**BMD Change (%), *m ± SD***	***P* value**
**12 months after transplantation**									
All, *n = 310*	−0.001 ± 0.063	−0.2 ± 6.3	0.793	−0.008 ± 0.054	−0.9 ± 7.3	0.034	−0.025 ± 0.051	−2.7 ± 5.8	< 0.001
Female, n = 116	+0.000 ± 0.055	+0.4 ± 5.8	0.996	−0.016 ± 0.058	−2.3 ± 8.2	0.068	−0.015 ± 0.051	−1.7 ± 6.4	0.005
< 50, *n = 59*	+0.006 ± 0.049	+1.0 ± 5.1	0.360	+0.007 ± 0.056	+0.9 ± 4.6	0.551	−0.002 ± 0.039	−0.2 ± 4.6	0.728
> 50, *n = 57*	−0.006 ± 0.061	−0.3 ± 6.5	0.450	−0.038 ± 0.051	−5.4 ± 7.6	0.001	−0.027 ± 0.059	−3.2 ± 7.5	0.002
Male, n = 194	−0.002 ± 0.068	−0.2 ± 6.5	0.754	−0.005 ± 0.052	−0.2 ± 6.7	0.359	−0.031 ± 0.050	−3.2 ± 5.4	< 0.001
< 50, *n = 79*	+0.005 ± 0.066	+1.0 ± 6.7	0.544	−0.006 ± 0.054	−0.3 ± 7.0	0.512	−0.030 ± 0.056	−3.0 ± 6.0	< 0.001
> 50, *n = 115*	−0.006 ± 0.068	−0.4 ± 6.3	0.377	−0.004 ± 0.052	−0.2 ± 6.6	0.525	−0.031 ± 0.045	−3.3 ± 5.0	< 0.001
ABD, n = 39	−0.014 ± 0.062	−0.9 ± 5.9	0.283	−0.025 ± 0.044	−3.3 ± 5.5	0.027	−0.048 ± 0.053	−4.9 ± 5.5	< 0.001
No ABD, n = 271	+0.000 ± 0.063	+0.4 ± 6.3	0.905	−0.006 ± 0.55	−0.6 ± 7.4	0.224	−0.022 ± 0.050	−2.4 ± 5.8	< 0.001
ESW, n = 41	+0.031 ± 0.066	+3.6 ± 6.6	0.006	+0.001 ± 0.056	+0.6 ± 8.1	0.909	−0.021 ± 0.061	−2.0 ± 6.7	0.047

OSR, n = 269	-0.006 ± 0.061	-0.2 ± 6.1	0.142	-0.011 ± 0.054	-1.2 ± 7.1	0.035	-0.026 ± 0.049	-2.8 ± 5.6	< 0.001
**24 months after transplantation**									
All, *n* = 222	−0.020 ± 0.066	−1.6 ± 6.4	< 0.001	−0.009 ± 0.059	−0.9 ± 7.7	0.045	−0.011 ± 0.062	−1.0 ± 6.9	0.017
Female, n = 86	−0.022 ± 0.061	−1.9 ± 6.2	0.001	−0.009 ± 0.064	−0.8 ± 8.9	0.407	−0.002 ± 0.057	−0.0 ± 7.0	0.756
< 50, *n = 46*	−0.018 ± 0.057	−1.6 ± 5.7	0.036	−0.013 ± 0.056	−1.7 ± 7.0	0.276	+0.006 ± 0.057	+0.9 ± 6.5	0.498
> 50, *n* = 40	−0.026 ± 0.066	−2.2 ± 6.7	0.015	−0.002 ± 0.075	+0.5 ± 11.1	0.896	−0.011 ± 0.056	−1.0 ± 7.5	0.249
Male, n = 136	−0.018 ± 0.069	−1.4 ± 6.5	0.003	−0.009 ± 0.056	−1.0 ± 7.1	0.196	−0.016 ± 0.065	−1.5 ± 6.9	0.008
< 50, *n = 60*	−0.011 ± 0.074	−0.4 ± 6.5	0.273	−0.004 ± 0.060	−0.1 ± 7.2	0.689	−0.007 ± 0.064	−0.3 ± 6.3	0.410
> 50, *n = 76*	−0.024 ± 0.065	−2.2 ± 6.5	0.002	−0.012 ± 0.054	−1.8 ± 7.0	0.167	−0.022 ± 0.065	−2.5 ± 7.2	0.005
ABD, n = 26	−0.039 ± 0.093	−3.0 ± 7.5	0.044	−0.012 ± 0.059	−2.1 ± 6.2	0.411	−0.034 ± 0.078	−3.2 ± 7.1	0.057
No ABD, n = 196	−0.017 ± 0.062	−1.5 ± 6.2	< 0.001	−0.008 ± 0.059	−0.7 ± 8.0	0.192	−0.008 ± 0.060	−0.7 ± 6.9	0.080
ESW, n = 16	+0.036 ± 0.059	+3.9 ± 5.8	0.028	+0.007 ± 0.064	+1.1 ± 9.5	0.702	+0.015 ± 0.056	+2.1 ± 7.0	0.275
OSR, n = 206	−0.024 ± 0.065	−2.0 ± 6.2	< 0.001	−0.010 ± 0.058	−1.2 ± 7.5	0.075	−0.013 ± 0.062	−1.2 ± 6.9	0.005

ABD, adynamic bone disease; BMD, bone mineral.

Table S3. Correlations between BMD at M1 and quantitative variables.

Quantitative Variables	Lumbar Spine BMD		Femoral Neck BMD		Total Hip BMD	
	ρ	*p* value	ρ	*p* value	ρ	*p* value
**Recipient's age**	+0.11	0.050	−0.12	0.078	−0.03	0.564
**BMI**	+0.31	<0.001	+0.27	<0.001	+0.42	<0.001
**Laboratory data (M0)**						
**Serum calcium**	−0.12	0.036	−0.06	0.403	−0.06	0.314
**Serum phosphate**	−0.04	0.451	−0.05	0.462	+0.02	0.679
**Serum 25 (OH) vitamin D3**	−0.04	0.463	−0.02	0.754	−0.04	0.485
**Serum PTH**	−0.17	0.003	−0.09	0.178	−0.13	0.040
**Serum bone alkaline phosphatases**	−0.05	0.453	−0.01	0.846	−0.04	0.557
**Serum osteocalcin**	−0.09	0.240	+0.06	0.456	−0.00	0.999

BMI, body mass index; ρ, Spearman's correlation coefficient; PTH, parathyroid hormone.

Table S4. Correlations between BMD at M1 and binary variables.

**Binary Variables**	**Lumbar Spine**			**Femoral Neck**			**Total Hip**		
	**BMD**		***p* value**	**BMD**		***p* value**	BMD		***p* value**
	No	Yes		No	Yes		No	Yes	
**Female**	1.034 ± 0.16	0.970 ± 0.16	<0.001	0.788 ± 0.16	0.693 ± 0.12	<0.001	0.929 ± 0.15	0.822 ± 0.13	<0.001
**Ethnic group (Caucasian)**	1.035 ± 0.16	1.008 ± 0.16	0.492	0.831 ± 0.22	0.751 ± 0.15	0.138	0.914 ± 0.13	0.890 ± 0.15	0.555
**Thyroid disorders**	1.009 ± 0.16	1.022 ± 0.17	0.750	0.758 ± 0.15	0.691 ± 0.10	0.122	0.893 ± 0.15	0.863 ± 0.15	0.454
**Prior osteoporotic fractures**	1.016 ± 0.16	1.004 ± 0.17	0.826	0.759 ± 0.15	0.705 ± 0.16	0.111	0.894 ± 0.15	0.860 ± 0.16	0.274
**Diabetes mellitus**	0.999 ± 0.16	1.065 ± 0.16	0.008	0.752 ± 0.15	0.766 ± 0.15	0.614	0.892 ± 0.15	0.885 ± 0.14	0.764
**Chronic inflammatory rheumatism**	1.011 ± 0.16	0.943 ± 0.16	0.402	0.755 ± 0.15	0.635 ± 0.09	0.262	0.893 ± 0.15	0.759 ± 0.09	0.079
**Autoimmune diseases**	1.014 ± 0.16	0.956 ± 0.16	0.121	0.756 ± 0.15	0.725 ± 0.17	0.482	0.894 ± 0.15	0.850 ± 0.17	0.247
**Primary HPT**	1.007 ± 0.16	1.130 ± 0.15	0.033	0.752 ± 0.15	0.815 ± 0.13	0.314	0.890 ± 0.15	0.932 ± 0.11	0.434
**Secondary HPT**	1.037 ± 0.16	1.005 ± 0.16	0.227	0.755 ± 0.14	0.754 ± 0.15	0.976	0.909 ± 0.15	0.888 ± 0.15	0.423
**Smoking**	1.003 ± 0.15	1.017 ± 0.17	0.461	0.745 ± 0.15	0.763 ± 0.15	0.382	0.882 ± 0.16	0.901 ± 0.15	0.284
**Alcohol consumption**	1.010 ± 0.16	1.015 ± 0.18	0.877	0.755 ± 0.15	0.732 ± 0.14	0.560	0.892 ± 0.15	0.877 ± 0.14	0.678
**Prior steroid intake**	1.024 ± 0.16	0.944 ± 0.15	<0.001	0.758 ± 0.14	0.732 ± 0.18	0.320	0.899 ± 0.14	0.852 ± 0.18	0.048
**Prior calcium intake**	0.999 ± 0.16	1.039 ± 0.17	0.052	0.746 ± 0.15	0.780 ± 0.16	0.155	0.885 ± 0.14	0.909 ± 0.7	0.234
**Prior vitamin D intake**	1.013 ± 0.15	1.007 ± 0.17	0.744	0.750 ± 0.13	0.758 ± 0.17	0.684	0.891 ± 0.14	0.891 ± 0.17	0.982
**Prior BP intake**	1.011 ± 0.16	0.864 ± 0.04	0.199	0.754 ± 0.15	0.750 ± 0.10	0.980	0.892 ± 0.15	0.810 ± 0.00	0.447

BP, bisphosphonate; HPT, hyperparathyroidism; MMF, mycophenolate mofetil;.

Table S5. Correlations between changes in BMD at M12 and quantitative variables.

Quantitative Variables	Lumbar Spine BMD		Femoral Neck BMD		Total Hip BMD	
	ρ	*p* value	ρ	*p* value	ρ	*p* value
**Recipient age**	−0.11	0.057	−0.10	0.225	−0.16	0.006
**BMI**	−0.11	0.064	+0.20	0.015	−0.02	0.706
**Laboratory data**						
**Serum calcium**	−0.01	0.892	+0.01	0.952	−0.11	0.074
**Serum phosphate**	+0.07	0.233	−0.03	0.703	−0.09	0.142
**Serum 25 (OH) vitamin D3**	−0.10	0.079	+0.03	0.759	−0.01	0.823
**Serum PTH**	+0.06	0.263	+0.20	0.019	+0.19	0.002
**Serum bone alkaline phosphatases**	+0.01	0.849	+0.19	0.038	+0.26	<0.001
**Serum osteocalcin**	+0.07	0.331	+0.14	0.175	+0.20	0.009
**Serum creatinine at M12**	−0.06	0.263	−0.09	0.258	+0.04	0.482

BMI, body mass index; ρ, Spearman's correlation coefficient; PTH, parathyroid hormone.

Table S6. Correlations between changes in BMD at M12 and binary variables.

Binary Variables	Lumbar Spine			Femoral Neck			Total Hip		
	BMD Variations		*p* value	BMD Variations		*p* value	BMD Variations		*p* value
	No	Yes		No	Yes		No	Yes	
**Female**	−0.002 ± 0.06	+0.000 ± 0.07	0.834	−0.005 ± 0.06	−0.016 ± 0.016	0.262	−0.015 ± 0.05	−0.031 ± 0.05	0.012
**Race (Caucasian)**	−0.002 ± 0.04	−0.001 ± 0.06	0.966	−0.002 ± 0.07	−0.009 ± 0.05	0.752	−0.001 ± 0.03	−0.026 ± 0.05	0.060
**Thyroid disorders**	−0.000 ± 0.06	+0.003 ± 0.04	0.327	−0.007 ± 0.05	−0.027 ± 0.06	0.309	−0.024 ± 0.05	−0.048 ± 0.04	0.086
**Prior osteoporotic fractures**	−0.00 ± 0.06	−0.009 ± 0.07	0.453	−0.009 ± 0.05	−0.005 ± 0.05	0.819	−0.025 ± 0.05	−0.023 ± 0.07	0.805
**Diabetes mellitus**	+0.001 ± 0.06	−0.008 ± 0.06	0.363	−0.010 ± 0.05	−0.005 ± 0.06	0.739	−0.025 ± 0.05	−0.026 ± 0.06	0.924
**Chronic inflammatory rheumatism**	−0.001 ± 0.06	+0.024 ± 0.06	0.431	−0.008 ± 0.05	−0.014 ± 0.01	0.893	−0.025 ± 0.05	−0.010 ± 0.03	0.552
**Autoimmune diseases**	−0.003 ± 0.06	+0.025 ± 0.06	0.054	−0.007 ± 0.05	−0.028 ± 0.08	0.229	−0.026 ± 0.05	−0.015 ± 0.04	0.383
**Primary HPT**	+0.001 ± 0.06	−0.077 ± 0.10	0.001	−0.006 ± 0.05	−0.117 ± 0.06	<0.001	−0.024 ± 0.05	−0.052 ± 0.06	0.155
**Secondary HPT**	−0.015 ± 0.07	+0.001 ± 0.06	0.105	−0.041 ± 0.06	−0.004 ± 0.05	0.005	−0.035 ± 0.05	−0.023 ± 0.05	0.182
**Smoking**	+0.000 ± 0.06	−0.002 ± 0.06	0.716	−0.002 ± 0.05	−0.015 ± 0.06	0.172	−0.024 ± 0.05	−0.026 ± 0.05	0.721
**Alcohol consumption**	−0.001 ± 0.06	−0.007 ± 0.06	0.649	−0.011 ± 0.06	−0.012 ± 0.04	0.852	−0.024 ± 0.05	−0.040 ± 0.05	0.175
**Calcium intake during the study period**	+0.005 ± 0.06	−0.012 ± 0.07	0.024	−0.010 ± 0.05	−0.005 ± 0.05	0.657	−0.026 ± 0.05	−0.024 ± 0.05	0.737
**Vitamin D intake during the study period**	−0.001 ± 0.07	−0.001 ± 0.06	0.966	+0.011 ± 0.06	−0.012 ± 0.05	0.074	−0.009 ± 0.06	−0.025 ± 0.05	0.113
**BP intake during the study period**	−0.003 ± 0.06	+0.054 ± 0.05	0.003	−0.008 ± 0.05	−0.036 ± 0.01	0.470	−0.025 ± 0.05	−0.028 ± 0.05	0.852
**Induction therapy**									
**Basiliximab**	−0.007 ± 0.07	+0.005 ± 0.06	0.107	−0.009 ± 0.05	−0.008 ± 0.06	0.961	−0.033 ± 0.05	−0.018 ± 0.05	0.014
**Thymoglobulin**	+0.004 ± 0.06	−0.007 ± 0.07	0.134	−0.008 ± 0.06	−0.009 ± 0.05	0.961	−0.019 ± 0.05	−0.032 ± 0.05	0.040
**Intravenous immunoglobulins**	−0.002 ± 0.06	+0.019 ± 0.08	0.241	−0.008 ± 0.05	−0.011 ± 0.04	0.879	−0.026 ± 0.05	−0.009 0.05	0.291
**Maintenance therapy**									

**MMF + tacrolimus**	−0.013 ± 0.06	+0.003 ± 0.06	0.064	+0.003 ± 0.06	−0.012 ± 0.05	0.178	−0.035 ± 0.05	−0.022 ± 0.05	0.062
**MMF + cyclosporine**	+0.006 ± 0.06	−0.014 ± 0.06	0.010	−0.012 ± 0.05	+0.001 ± 0.05	0.222	−0.022 ± 0.05	−0.031 ± 0.05	0.182
**Tacrolimus + everolimus**	+0.000 ± 0.06	−0.011 ± 0.07	0.377	−0.008 ± 0.05	−0.015 ± 0.05	0.704	−0.025 ± 0.05	−0.025 ± 0.05	0.994
**MMF + everolimus**	−0.001 ± 0.06	−0.003 ± 0.06	0.836	−0.006 ± 0.06	−0.022 ± 0.04	0.246	−0.023 ± 0.05	−0.037 ± 0.05	0.095
**Tacrolimus + azathioprine**	−0.001 ± 0.06	+0.004 ± 0.770	0.770	−0.008 ± 0.05	−0.028 ± 0.01	0.476	−0.026 ± 0.05	−0.008 ± 0.04	0.206
**Early steroid withdrawal**	−0.006 ± 0.06	+0.031 ± 0.07	<0.001	−0.010 ± 0.05	+0.001 ± 0.06	0.318	−0.026 ± 0.05	−0.021 ± 0.06	0.583

BP, bisphosphonate; HPT, hyperparathyroidism; MMF, mycophenolate mofetil.

Table S7. Correlations between changes in BMD at M24 and quantitative variables.

Quantitative Variables	Lumbar spine		Femoral neck		Total hip	
	Correlation coefficient	*P* value	Correlation coefficient	*P* value	Correlation coefficient	*P* value
**Recipient age**	−0.07	0.311	−0.08	0.397	−0.19	0.006
**BMI**	+0.00	0.973	+0.09	0.326	−0.06	0.398
**Laboratory data**						
**Serum calcium**	+0.09	0.172	+0.06	0.551	−0.03	0.658
**Serum phosphate**	+0.14	0.033	+0.24	0.011	+0.01	0.856
**Serum 25 (OH) vitamin D3**	−0.08	0.246	+0.06	0.563	+0.00	0.987
**Serum PTH**	+0.07	0.302	+0.28	0.003	+0.19	0.006
**Serum bone alkaline phosphatases**	+0.01	0.866	+0.08	0.449	+0.22	0.005
**Serum osteocalcin**	+0.14	0.099	+0.28	0.017	+0.19	0.061
**Serum creatinine at M12**	−0.13	0.048	−0.28	0.003	−0.15	0.028

BMI, body mass index; ρ, Spearman’s correlation coefficient; PTH, parathyroid hormone.

Table S8. Correlations between changes in BMD at M24 and binary variables.

Binary Variables	Lumbar Spine			Femoral Neck			Total Hip		
	BMD Variations		*p* value	BMD Variations		*p* value	BMD Variations		*p* value
	No	Yes		No	Yes		No	Yes	
**Female**	−0.018 ± 0.06	−0.022 ± 0.06	0.662	−0.009 ± 0.06	−0.009 ± 0.06	0.992	−0.002 ± 0.06	−0.016 ± 0.06	0.140
**Race (Caucasian)**	−0.024 ± 0.07	−0.019 ± 0.07	0.774	−0.017 ± 0.03	−0.008 ± 0.06	0.721	−0.001 ± 0.04	−0.011 ± 0.06	0.602
**Thyroid disorders**	−0.019 ± 0.07	−0.020 ± 0.04	0.977	−0.007 ± 0.06	−0.039 ± 0.05	0.236	−0.010 ± 0.06	−0.023 ± 0.03	0.483
**Prior osteoporotic fractures**	−0.021 ± 0.07	−0.003 ± 0.06	0.236	−0.009 ± 0.05	−0.009 ± 0.09	0.786	−0.012 ± 0.06	+0.001 ± 0.06	0.401
**Diabetes mellitus**	−0.018 ± 0.07	−0.027 ± 0.07	0.480	−0.005 ± 0.06	−0.041 ± 0.05	0.052	−0.009 ± 0.06	−0.019 ± 0.06	0.490
**Chronic inflammatory rheumatism**	−0.021 ± 0.07	+0.022 ± 0.06	0.203	−0.008 ± 0.06	−0.019 ± 0.03	0.802	−0.011 ± 0.06	−0.011 ± 0.02	0.984
**Autoimmune diseases**	−0.022 ± 0.07	+0.015 ± 0.06	0.031	−0.009 ± 0.06	−0.005 ± 0.09	0.869	−0.012 ± 0.06	+0.013 ± 0.06	0.134
**Primary HPT**	−0.017 ± 0.06	−0.098 ± 0.010	0.003	−0.008 ± 0.06	−0.021 ± 0.09	0.664	−0.010 ± 0.06	−0.032 ± 0.05	0.391
**Secondary HPT**	−0.029 ± 0.08	−0.018 ± 0.06	0.331	−0.029 ± 0.06	−0.005 ± 0.06	0.131	−0.016 ± 0.06	−0.010 ± 0.06	0.602
**Smoking**	−0.020 ± 0.07	−0.019 ± 0.06	0.894	−0.012 ± 0.05	−0.005 ± 0.07	0.562	−0.011 ± 0.07	−0.010 ± 0.05	0.859
**Alcohol consumption**	−0.019 ± 0.07	−0.020 ± 0.05	0.964	−0.009 ± 0.06	+0.008 ± 0.05	0.495	−0.010 ± 0.06	−0.020 ± 0.07	0.613
**Prior steroid intake**	−0.021 ± 0.06	−0.013 ± 0.08	0.523	−0.009 ± 0.06	−0.008 ± 0.05	0.950	−0.013 ± 0.016	+0.002 ± 0.07	0.173
**Calcium intake during the study period**	−0.015 ± 0.06	−0.027 ± 0.08	0.197	−0.005 ± 0.06	−0.015 ± 0.06	0.388	−0.010 ± 0.06	−0.012 ± 0.06	0.782

**Vitamin D intake during the study period**	−0.017 ± 0.06	−0.020 ± 0.07	0.854	−0.019 ± 0.06	−0.007 ± 0.06	0.442	+0.009 ± 0.05	−0.014 ± 0.06	0.089
**BP intake during the study period**	−0.023 ± 0.07	+0.053 ± 0.03	<0.001	−0.010 ± 0.06	+0.043 ± 0.10	0.076	−0.011 ± 0.06	−0.003 ± 0.05	0.715
**Induction therapy**									
**Basiliximab**	−0.024 ± 0.07	−0.015 ± 0.06	0.300	−0.002 ± 0.05	−0.013 ± 0.06	0.331	−0.010 ± 0.05	−0.011 ± 0.07	0.924
**Thymoglobulin**	−0.015 ± 0.06	−0.024 ± 0.07	0.289	−0.013 ± 0.06	−0.002 ± 0.05	0.331	−0.012 ± 0.07	−0.010 ± 0.06	0.795
**Intravenous immunoglobulins**	−0.020 ± 0.07	−0.012 ± 0.04	0.783	−0.008 ± 0.06	−0.049 ± 0.02	0.330	−0.010 ± 0.06	−0.021 ± 0.05	0.664
**Maintenance therapy**									
**MMF + tacrolimus**	−0.022 ± 0.05	−0.017 ± 0.07	0.748	−0.006 ± 0.05	−0.010 ± 0.06	0.742	−0.021 ± 0.06	−0.007 ± 0.06	0.148
**MMF + cyclosporine**	−0.015 ± 0.07	−0.027 ± 0.06	0.210	−0.009 ± 0.06	−0.008 ± 0.05	0.960	−0.006 ± 0.06	−0.018 ± 0.06	0.193
**Tacrolimus + everolimus**	−0.020 ± 0.07	−0.020 ± 0.05	1.000	−0.009 ± 0.06	−0.006 ± 0.05	0.871	−0.009 ± 0.06	−0.024 ± 0.07	0.315
**MMF + everolimus**	−0.020 ± 0.07	−0.020 ± 0.0	0.951	−0.012 ± 0.06	+0.008 ± 0.04	0.154	−0.010 ± 0.06	−0.014 ± 0.06	0.715
**Tacrolimus + azathioprine**	−0.021 ± 0.07	−0.002 ± 0.06	0.347	−0.009 ± 0.06	−0.008 ± 0.03	0.976	−0.012 ± 0.06	−0.007 ± 0.04	0.301
**Early steroid withdrawal**	−0.024 ± 0.06	+0.036 ± 0.06	**<0.001**	−0.010 ± 0.06	+0.007 ± 0.06	0.328	−0.013 ± 0.06	+0.016 ± 0.06	0.077

BP, bisphosphonate; HPT, hyperparathyroidism; MMF, mycophenolate mofetil;.

Table S9. Predictive factors for a BMD decrease at M1 (multiple linear regression model).

BMD at M1	Coefficient	T value	*P* value
**Lumbar spine**			
**IxS**	$+5.555 \times 10^{-4}$	+1.167	0.245
**BMI**	$+5.153 \times 10^{-3}$	+4.001	<0.001
**Male (ref. = female)**	+0.035	+3.174	0.002
**Serum calcium**	−0.075	−2.774	0.006
**Serum phosphate**	-2.248×10^{-4}	−0.019	0.985
**Serum 25 (OH) vitamin D3**	-3.740×10^{-5}	−0.121	0.904
**Serum PTH**	-3.815×10^{-5}	−2.494	0.014
**Serum bone alkaline phosphatases**	7.782×10^{-5}	+0.176	0.861
**Serum osteocalcin**	-8.958×10^{-6}	−0.446	0.656
**Femoral neck**			
**IxS**	+0.001	+2.063	0.042
**BMI**	+0.005	+3.280	0.001
**Male (ref. = female)**	+0.038	+2.964	0.004
**Serum calcium**	−0.049	−1.599	0.112
**Serum phosphate**	−0.011	−0.714	0.477
**Serum 25 (OH) vitamin D3**	-3.310×10^{-4}	−0.953	0.342
**Serum PTH**	-2.753×10^{-5}	−1.346	0.181
**Serum bone alkaline phosphatases**	$+1.221 \times 10^{-4}$	+0.234	0.815
**Serum osteocalcin**	$+1.942 \times 10^{-5}$	+0.849	0.397
**Total hip**			
**IxS**	$+6.276 \times 10^{-4}$	+1.396	0.164
**BMI**	$+6.001 \times 10^{-3}$	+4.998	<0.001
**Male (ref. = female)**	$+4.558 \times 10^{-2}$	+4.379	<0.001
**Serum calcium**	-7.103×10^{-2}	−2.880	0.005
**Serum phosphate**	$+1.731 \times 10^{-3}$	+0.155	0.878
**Serum 25 (OH) vitamin D3**	-2.474×10^{-4}	−0.811	0.418
**Serum PTH**	-3.054×10^{-5}	−1.935	0.054
**Serum bone alkaline phosphatases**	$+1.853 \times 10^{-4}$	+0.391	0.697
**Serum osteocalcin**	$+4.547 \times 10^{-6}$	+0.251	0.802

BMI, body mass index; IxS, indoxylsulfate;.

Table S10. Correlations between UT concentrations and BMD at M1, by subgroup.

	**Uremic Toxins**													
	**pCS**		**CMPF**		**IxS**		**pCG**		**HA**		**TMAO**		**IAA**	
**Subgroups**	**r**	***p* value**	**r**	***p* value**	**r**	***p* value**	**r**	***p* value**	**r**	***p* value**	**r**	***p* value**	**r**	***p* value**
**Lumbar spine**														
**All, *n* = *310***	+0.02	0.782	+0.01	0.805	+0.04	0.477	−0.01	0.876	−0.06	0.300	+0.01	0.797	+0.03	0.656
Female, n* = *116	−0.07	0.485	−0.08	0.417	+0.10	0.271	−0.15	0.111	−0.19	0.038	−0.03	0.744	+0.04	0.689
**< 50, *n* = *59***	−0.01	0.939	−0.01	0.956	+0.19	0.144	+0.02	0.863	−0.10	0.494	−0.04	0.752	+0.06	0.636
**> 50, *n* = 57**	−0.08	0.542	−0.22	0.095	−0.04	0.796	−0.26	0.049	−0.30	0.022	−0.04	0.744	−0.01	0.959
Male, n* = *194	+0.03	0.710	+0.06	0.408	−0.04	0.565	+0.07	0.364	−0.02	0.802	+0.03	0.700	−0.01	0.937
**< 50, *n* = *79***	−0.03	0.814	+0.04	0.698	−0.04	0.758	+0.02	0.873	−0.00	0.974	+0.08	0.493	−0.18	0.107
**> 50, *n* = *115***	+0.07	0.478	+0.03	0.752	−0.02	0.803	+0.10	0.311	−0.03	0.722	−0.00	0.989	+0.03	0.750
ABD, n* = *39	+0.03	0.852	−0.33	0.038	+0.31	0.055	+0.16	0.341	+0.14	0.393	−0.07	0.666	+0.08	0.608
No ABD, n* = *271	60.01	0.834	+0.05	0.369	+0.01	0.877	−0.04	0.551	−0.07	0.238	+0.03	0.634	+0.02	0.799
**Femoral neck**														
**All, *n* = *310***	+0.02	0.730	−0.05	0.448	+0.13	0.049	+0.03	0.701	−0.14	0.036	−0.00	0.975	−0.07	0.282
Female, n* = *116	−0.12	0.275	+0.05	0.641	+0.12	0.282	−0.12	0.288	−0.15	0.180	−0.04	0.734	+0.07	0.540
**< 50, *n* = *59***	−0.03	0.838	+0.18	0.249	+0.10	0.538	−0.10	0.543	−0.17	0.268	+0.06	0.722	+0.09	0.584
**> 50, *n* = 57**	−0.23	0.165	−0.17	0.299	+0.14	0.398	−0.14	0.396	−0.13	0.442	−0.16	0.344	+0.05	0.776
Male, n* = *194	+0.03	0.759	−0.12	0.155	+0.07	0.394	+0.03	0.691	−0.18	0.035	+0.02	0.811	−0.16	0.058
**< 50, *n* = *79***	−0.12	0.372	−0.18	0.177	+0.21	0.108	+0.01	0.942	−0.14	0.305	+0.10	0.463	−0.22	0.097
**> 50, *n* = *115***	+0.14	0.197	−0.06	0.585	−0.10	0.381	+0.05	0.641	−0.21	0.061	−0.04	0.719	−0.15	0.161
ABD, n* = *39	+0.09	0.623	−0.32	0.075	+0.30	0.112	+0.06	0.772	+0.00	0.981	−0.27	0.146	−0.17	0.377

No ABD, n = 271	+0.01	0.893	−0.01	0.912	+0.10	0.174	+0.01	0.904	−0.16	0.026	+0.06	0.368	−0.05	0.455
**Total hip BMD**														
**All, *n* = *310***	−0.01	0.913	+0.03	0.592	+0.13	0.037	+0.01	0.901	−0.08	0.190	−0.00	0.942	−0.02	0.738
Female, n = 116	−0.23	0.024	+0.11	0.281	+0.18	0.068	−0.13	0.191	−0.13	0.190	−0.08	0.416	+0.09	0.388
**< 50, *n* = *59***	−0.11	0.441	+0.20	0.165	+0.16	0.261	+0.02	0.916	−0.08	0.568	−0.07	0.611	+0.13	0.369
**> 50, *n* = 57**	−0.30	0.034	−0.07	0.633	+0.15	0.298	−0.25	0.082	−0.22	0.119	−0.13	0.356	−0.04	0.797
Male, n = 194	+0.04	0.563	−0.04	0.555	+0.01	0.876	+0.07	0.355	−0.12	0.112	+0.01	0.931	−0.09	0.232
**< 50, *n* = *79***	+0.04	0.753	−0.02	0.853	+0.07	0.554	+0.03	0.776	−0.11	0.349	+0.02	0.892	−0.12	0.312
**> 50, *n* = *115***	+0.05	0.631	−0.05	0.620	−0.05	0.618	+0.10	0.319	−0.12	0.200	−0.01	0.892	−0.09	0.367
ABD, n = 39	+0.19	0.297	−0.19	0.291	+0.34	0.055	+0.25	0.165	+0.17	0.337	−0.20	0.267	−0.06	0.728
No ABD, n = 271	−0.05	0.490	+0.07	0.267	+0.09	0.162	−0.03	0.610	−0.10	0.106	+0.04	0.568	−0.01	0.830

ABD, adynamic bone disease; CMPF, 3-carboxy-4-methyl-5-propyl-furanpropionic acid; HA, hippuric acid; IAA, indole-3-acetic acid; IxS, indoxylsulfate; pCG, p-cresylglucuronide; pCS, p-cresylsulfate; r, correlation coefficient; TMAO, trimethylamine-N-oxide.

Table S11. Comparison of UT concentrations in patients with BMD loss vs. gain 12 months after kidney transplantation, by subgroup.

**Subgroups**	**Lumbar Spine**			**Femoral Neck**			**Total Hip**		
	**BMD loss *n* = 154**	**BMD gain *n* = 156**	***p***	**BMD loss *n* = 89**	**BMD gain *n* = 56**	***p***	**BMD loss *n* = 197**	**BMD gain *n* = 79**	***p***
**pCS (μg/mL)**									
**All, *n* = *310***	16.2 [0.02–65.7]	15.9 [0.3–68.1]	0.857	13.9 [0.1–52.5]	15.4 [0.5–65.7]	0.556	16.0 [0.1–68.1]	16.0 [0.6–57.3]	0.964
Female, n = 116	14.1 [0.5–57.3]	14.5 [1.8–46.8]	0.790	13.3 [0.6–42.8]	9.6 [0.5–30.6]	0.515	13.2 [0.6–48.47]	15.3 [1.9–57.3]	0.354
**< 50, *n* = *59***	11.5 [0.5–48.7]	12.2 [1.8–48.8]	0.727	8.4 [6.6–48.8]	9.6 [0.5–30.6]	0.487	11.2 [0.7–48.7]	14.6 [1.9–45.4]	0.240
**> 50, *n* = 57**	16.7 [0.6–57.3]	17.2 [3.4–46.8]	0.806	14.1 [0.6–33.5]	13.9 [3.4–23.8]	0.561	16.4 [0.6–43.8]	16.8 [6.9–57.3]	0.667

Male, n* = *194	17.4 [0.02–65.7]	16.9 [0.3–68.1]	0.912	13.9 [0.1–52.5]	13.4 [0.6–65.7]	0.740	17.0 [0.1–68.1]	16.9 [0.6–55.6]	0.732
**< 50, *n* = *79***	16.7 [0.02–46.2]	18.5 [0.3–68.1]	0.432	17.4 [0.3–48.8]	15.5 [0.6–49.5]	0.967	16.4 [0.3–68.1]	19.5 [0.6–49.5]	0.489
**> 50, *n* = *115***	17.7 [0.07–65.7]	16.5 [0.3–55.6]	0.700	13.9 [0.1–52.5]	13.3 [3.2–65.7]	0.701	17.1 [0.1–54.0]	13.2 [3.2–55.6]	0.262
ABD, n* = *39	16.7 [0.1–34.7]	17.6 [3.4–52.5]	0.254	14.6 [7.6–52.5]	13.3 [3.4–23.7]	0.336	14.3 [0.8–52.2]	21.5 [13.3–42.8]	0.281
No ABD, n* = *271	16.0 [0.02–65.7]	15.9 [0.3–68.1]	0.901	13.0 [0.1–48.8]	13.4 [0.5–65.7]	0.838	16.4 [0.1–68.1]	15.7 [0.6–57.3]	0.848
GFR > 60 mL/min, n* = *102	18.1 [0.02–50.6]	14.8 [1.01–50.8]	0.528	16.7 [1.1–46.2]	11.9 [0.5–32.7]	0.552	17.0 [1.1–50.8]	15.1 [1.9–42.8]	0.935
GFR < 60 mL/min, n* = *208	18.6 [0.1–65.7]	16.1 [0.3–68.1]	0.504	13.5 [0.1–52.5]	13.4 [0.6–65.7]	0.763	15.5 [0.1–68.1]	16.1 [0.6–57.3]	0.935
**CMPF (μg/mL)**									
**All, *n* = *310***	3.0 [0.0–32.3]	2.0 [0.0–18.0]	0.005	2.9 [0.0–22.0]	2.2 [0.0–32.3]	0.070	2.5 [0.0–28.3]	2.4 [0.0–18.0]	0.921
Female, n* = *116	2.4 [0.0–32.3]	1.9 [0.0–12.2]	0.437	2.2 [0.1–22.0]	1.5 [0.0–32.3]	0.098	1.6 [0.0–22.0]	2.4 [0.1–14.0]	0.266
**< 50, *n* = *59***	2.9 [0.0–5.2]	3.3 [0.0–12.2]	0.767	5.0 [0.8–22.0]	2.4 [0.0–32.3]	0.051	3.1 [0.0–22.0]	3.6 [0.1–14.0]	0.330
**> 50, *n* = 57**	1.9 [0.1–12.2]	1.2 [0.2–12.2]	0.154	2.9 [0.1–12.2]	1.1 [0.3–1.5]	0.152	1.3 [0.1–12.2]	1.0 [0.2–8.4]	1.000
Male, n* = *194	3.1 [0.0–23.8]	2.1 [0.0–18.0]	0.004	3.3 [0.0–14.1]	2.4 [0.0–18.0]	0.254	2.7 [0.0–28.3]	2.4 [0.0–18.0]	0.603
**< 50, *n* = *79***	2.6 [0.0–19.0]	1.8 [0.0–8.6]	0.364	2.0 [0.0–9.7]	2.4 [0.0–7.3]	0.814	2.1 [0.0–19.0]	2.5 [0.0–7.3]	0.722
**> 50, *n* = *115***	4.5 [0.1–28.3]	2.6 [0.0–18.0]	0.004	4.4 [0.3–14.1]	2.4 [0.0–18.0]	0.212	3.3 [0.0–28.3]	2.4 [0.1–18.0]	0.462
ABD, n* = *39	1.9 [0.0–12.2]	1.3 [0.0–7.4]	0.602	2.8 [0.0–12.2]	1.0 [0.0–2.7]	0.104	1.5 [0.0–7.4]	1.1 [0.4–5.9]	1.000
No ABD, n* = *271	3.2 [0.0–32.3]	2.1 [0.0–18.0]	0.003	3.0 [0.0–22.0]	2.4 [0.0–32.3]	0.154	2.6 [0.0–28.3]	2.4 [0.0–18.0]	0.739

GFR > 60 mL/min, n = 102	2.6<br>[0.0–32.3]	2.8<br>[0.0–12.2]	0.56<br>0	2.9<br>[0.1–22.0]	1.4<br>[0.0–32.3]	0.07<br>9	2.6<br>[0.0–22.0]	2.5<br>[0.1–14.0]	0.872
GFR < 60 mL/min, n = 208	3.4<br>[0.0–28.3]	1.8<br>[0.0–18.0]	0.00<br>3	3.3<br>[0.0–14.1]	2.4<br>[0.0–18.0]	0.34<br>5	2.4<br>[0.0–28.3]	2.3<br>[0.0–18.0]	0.997
**IxS (µg/mL)**									
**All, *n* = 310**	20.4<br>[1.2–101.0]	19.6<br>[1.8–67.2]	0.40<br>1	19.3<br>[1.8–68.6]	19.9<br>[1.3–54.5]	0.86<br>1	19.8<br>[1.2–67.2]	20.7<br>[3.7–101.0]	0.476
Female, n = 116	16.4<br>[1.2–101.0]	16.1<br>[3.7–34.8]	0.81<br>6	15.0<br>[2.2–34.8]	18.1<br>[6.8–40.8]	0.39<br>2	14.6<br>[1.2–57.7]	18.6<br>[3.7–101.0]	0.027
**< 50, *n* = 59**	19.4<br>[3.4–101.0]	17.5<br>[6.8–27.8]	0.49<br>9	16.7<br>[9.3–34.8]	18.1<br>[6.8–40.8]	0.92<br>8	16.8<br>[3.4–57.7]	18.2<br>[8.1–101.0]	0.252
**> 50, *n* = 57**	14.2<br>[1.2–49.5]	15.0<br>[3.7–31.1]	0.74<br>9	13.4<br>[2.2–33.3]	17.3<br>[9.6–30.7]	0.68<br>2	12.6<br>[1.2–33.3]	19.9<br>[3.7–31.1]	0.164
Male, n = 194	21.3<br>[1.3–68.6]	20.7<br>[1.8–67.2]	0.53<br>0	23.7<br>[1.8–68.6]	20.0<br>[1.3–54.5]	0.61<br>9	21.2<br>[1.3–67.2]	22.1<br>[3.8–54.5]	0.982
**< 50, *n* = 79**	22.4<br>[2.8–68.6]	25.89<br>[4.2–67.2]	0.70<br>2	21.4<br>[3.2–68.6]	25.9<br>[7.6–40.1]	0.78<br>3	23.1<br>[3.2–67.2]	27.4<br>[6.2–40.1]	0.752
**> 50, *n* = 115**	20.6<br>[1.3–55.1]	19.8<br>[1.8–65.4]	0.26<br>9	19.8<br>[1.8–54.6]	17.5<br>[1.3–54.5]	0.41<br>4	20.6<br>[1.3–65.4]	18.4<br>[3.8–54.5]	0.697
ABD, n = 39	16.0<br>[3.4–65.3]	16.2<br>[3.7–54.6]	0.72<br>4	17.7<br>[6.6–54.6]	17.0<br>[8.5–38.2]	0.77<br>5	16.2<br>[3.4–56.3]	17.0<br>[3.7–34.8]	0.514
No ABD, n = 271	20.6<br>[1.2–101.0]	19.9<br>[1.8–67.2]	0.39<br>8	19.6<br>[1.8–68.6]	20.0<br>[1.3–54.5]	0.79<br>8	20.0<br>[1.2–67.2]	21.2<br>[4.8–101.0]	0.439
GFR > 60 mL/min, n = 102	22.6<br>[2.8–101.0]	17.5<br>[3.7–66.7]	0.01<br>8	15.5<br>[3.2–68.6]	18.4<br>[7.5–38.7]	0.63<br>0	20.2<br>[3.2–66.7]	17.8<br>[3.7–101.0]	0.553
GFR < 60 mL/min, n = 208	19.3<br>[1.2–62.0]	20.7<br>[1.8–67.2]	0.53<br>2	19.8<br>[1.8–62.0]	20.0<br>[1.3–54.5]	0.98<br>8	19.3<br>[1.2–67.2]	21.4<br>[3.8–54.5]	0.221
**pCG (µg/mL)**									
**All, *n* = 310**	0.89<br>[0.00–9.10]	0.71<br>[0.00–6.80]	0.36<br>8	0.72<br>[0.00–7.04]	0.66<br>[0.00–5.75]	0.48<br>8	0.80<br>[0.00–9.10]	0.69<br>[0.00–5.17]	0.786
Female, n = 116	0.84<br>[0.00–6.89]	0.60<br>[0.00–4.19]	0.43<br>1	0.56<br>[0.00–3.05]	0.60<br>[0.00–2.75]	0.99<br>1	0.79<br>[0.00–6.89]	0.62<br>[0.00–5.17]	0.833
**< 50, *n* = 59**	0.77<br>[0.00–1.46]	0.55<br>[0.00–1.48]	0.61<br>1	0.52<br>[0.00–2.88]	0.98<br>[0.00–2.75]	0.51<br>7	0.53<br>[0.00–6.89]	0.67<br>[0.00–2.88]	0.992

**> 50, *n* = 57**	0.87 [0.00–5.17]	0.87 [0.00–4.19]	0.605	0.61 [0.00–3.10]	0.37 [0.13–0.84]	0.295	0.84 [0.00–6.22]	0.56 [0.00–5.17]	0.894
***Male, n* = 194**	0.90 [0.00–9.10]	0.75 [0.00–6.80]	0.576	0.79 [0.00–7.04]	0.69 [0.00–5.75]	0.312	0.80 [0.00–9.10]	0.78 [0.00–5.14]	0.772
**< 50, *n* = 79**	0.72 [0.00–4.51]	0.76 [0.00–6.80]	0.672	0.12 [0.00–6.80]	0.85 [0.00–4.38]	0.751	0.79 [0.00–6.80]	0.64 [0.00–4.38]	0.930
**> 50, *n* = 115**	0.97 [0.00–9.10]	0.69 [0.00–4.27]	0.293	0.94 [0.00–7.04]	0.69 [0.00–5.75]	0.275	0.80 [0.00–9.10]	0.90 [0.00–5.14]	0.651
***ABD, n* = 39**	0.81 [0.00–7.04]	1.03 [0.00–3.99]	0.782	0.90 [0.00–7.04]	0.93 [0.00–1.58]	0.489	0.79 [0.00–7.04]	0.93 [0.06–2.88]	0.403
***No ABD, n* = 271**	0.90 [0.00–9.10]	0.70 [0.00–6.80]	0.336	0.67 [0.00–6.80]	0.63 [0.00–5.75]	0.701	0.80 [0.00–9.10]	0.67 [0.00–5.17]	0.606
***GFR > 60 mL/min, n* = 102**	0.70 [0.00–6.89]	0.72 [0.00–4.65]	0.492	0.61 [0.00–3.53]	0.98 [0.00–5.75]	0.894	0.76 [0.00–6.89]	0.60 [0.00–3.84]	0.239
***GFR < 60 mL/min, n* = 208**	0.92 [0.00–9.10]	0.71 [0.00–6.80]	0.543	0.82 [0.00–7.04]	0.58 [0.00–5.14]	0.375	1.31 [0.00–9.1]	1.29 [0.00–5.17]	0.744
**HA (μg/mL)**									
**All, *n* = 310**	25.6 [0.4–195.0]	25.7 [1.0–139.0]	0.842	28.0 [1.8–139.0]	25.7 [0.4–100.0]	0.932	24.0 [0.4–195.0]	30.7 [1.1–100.0]	0.471
***Female, n* = 116**	20.5 [1.8–100.0]	22.4 [1.1–139.0]	0.687	22.2 [1.8–139.0]	34.4 [4.1–80.8]	0.451	19.1 [1.8–139.0]	30.7 [1.1–98.7]	0.184
**< 50, *n* = 59**	17.1 [2.0–100.0]	24.0 [1.1–139.0]	0.682	22.2 [2.0–139.0]	50.8 [4.04–80.8]	0.325	19.7 [2.0–139.0]	24.9 [1.1–98.7]	0.574
**> 50, *n* = 57**	23.3 [1.8–83.2]	20.8 [1.1–100.0]	0.812	25.0 [1.8–100.0]	17.5 [5.9–34.4]	0.477	18.5 [1.8–117.0]	34.0 [1.1–90.1]	0.199
***Male, n* = 194**	28.1 [0.4–195.0]	29.1 [1.0–100.0]	0.869	29.1 [1.8–124.0]	25.6 [0.4–100.0]	0.494	28.4 [0.4–195.0]	30.6 [2.9–100.0]	0.821
**< 50, *n* = 79**	22.0 [0.5–195.0]	25.7 [1.0–100.0]	0.541	22.4 [1.8–124.0]	25.7 [2.9–78.6]	0.946	22.8 [0.5–195.0]	25.7 [2.9–100.0]	0.697
**> 50, *n* = 115**	38.4 [0.4–126.0]	36.1 [2.4–100.0]	0.486	37.0 [2.4–63.4]	19.1 [7.0–36.9]	0.246	38.4 [0.4–126.0]	37.3 [3.4–76.7]	0.954
***ABD, n* = 39**	25.8 [3.2–82.2]	20.6 [1.1–63.4]	0.323	24.7 [1.8–139.0]	25.7 [0.4–100.0]	0.871	24.5 [3.2–82.2]	19.1 [1.1–24.0]	0.129

No ABD, n = 271	25.3 [0.4–195.0]	27.3 [1.0–139.0]	0.661	24.6 [1.8–139.0]	21.1 [3.36–98.4]	0.766	24.0 [0.4–195.0]	32.8 [1.1–100.0]	0.315
GFR > 60 mL/min, n = 102	23.1 [1.8–100.0]	21.3 [1.0–139.0]	0.769	30.0 [1.8–124.0]	33.6 [0.4–100.0]	0.863	21.4 [1.0–139.0]	21.1 [1.1–100.0]	0.872
GFR < 60 mL/min, n = 208	28.2 [0.4–195.0]	29.9 [2.4–100.0]	0.622	28.2 [0.4–195.0]	29.9 [2.4–100.0]	0.622	25.0 [0.4–195.0]	33.9 [2.9–94.3]	0.274
**TMAO (µg/mL)**									
**All, *n* = 310**	4.4 [0.0–32.5]	4.2 [0.0–54.0]	0.577	4.2 [0.0–22.7]	4.1 [0.0–32.5]	0.534	4.3 [0.0–54.0]	4.5 [0.0–32.4]	0.433
Female, n = 116	3.3 [0.0–31.6]	4.2 [0.0–34.1]	0.525	3.3 [0.0–15.2]	3.8 [0.0–14.7]	0.515	3.5 [0.0–31.6]	5.1 [0.0–32.4]	0.110
**< 50, *n* = 59**	3.2 [0.0–29.0]	4.8 [0.1–34.1]	0.103	3.7 [0.1–15.2]	3.8 [0.0–14.7]	0.786	3.5 [0.1–15.2]	7.6 [0.5–32.4]	0.006
**> 50, *n* = 57**	3.5 [0.0–31.6]	3.3 [0.0–15.4]	0.385	3.2 [0.0–12.7]	3.7 [0.4–9.7]	0.969	4.2 [0.0–31.6]	3.4 [0.0–7.7]	0.519
Male, n = 194	5.1 [0.0–32.5]	4.3 [0.0–54.0]	0.221	4.4 [0.0–22.7]	4.3 [0.0–32.5]	0.963	4.7 [0.0–54.0]	4.4 [0.0–20.5]	0.954
**< 50, *n* = 79**	4.3 [0.0–29.1]	4.5 [0.8–54.0]	0.850	3.8 [0.8–22.7]	3.7 [1.6–14.9]	0.825	4.4 [0.0–54.0]	3.7 [1.6–16.1]	0.778
**> 50, *n* = 115**	5.9 [0.0–32.5]	4.2 [0.0–24.0]	0.058	5.0 [0.0–18.1]	4.6 [0.0–32.5]	0.766	4.7 [0.0–32.5]	4.5 [0.0–20.5]	0.979
ABD, n = 39	4.2 [0.0–31.6]	4.1 [0.4–12.3]	0.866	5.4 [0.0–12.3]	3.3 [0.4–4.6]	0.059	4.2 [0.0–31.6]	4.5 [0.4–12.3]	0.610
No ABD, n = 271	4.5 [0.0–32.5]	4.3 [0.0–54.0]	0.609	4.0 [0.0–22.7]	4.3 [0.0–32.5]	0.190	4.5 [0.0–54.0]	4.4 [0.0–32.4]	0.495
GFR > 60 mL/min, n = 102	4.0 [0.0–29.2]	4.8 [0.1–22.7]	0.918	4.1 [0.1–22.7]	4.3 [0.0–14.9]	0.831	4.0 [0.0–22.7]	5.8 [0.4–29.2]	0.090
GFR < 60 mL/min, n = 208	4.9 [0.0–32.5]	4.1 [0.0–54.0]	0.398	4.2 [0.0–17.1]	3.9 [0.0–32.5]	0.552	4.5 [0.0–54.0]	3.8 [0.0–32.4]	0.807
**IAA (µg/mL)**									
**All, *n* = 310**	0.75 [0.08–7.61]	0.75 [0.10–5.28]	0.302	0.74 [0.14–7.61]	0.75 [0.24–5.89]	0.940	0.74 [0.19–7.61]	0.78 [0.08–5.89]	0.808
Female, n = 116	0.69 [0.19–5.22]	0.77 [0.10–2.83]	0.876	0.67 [0.14–2.52]	0.71 [0.35–1.80]	0.627	0.71 [0.19–5.22]	0.78 [0.14–2.52]	0.365

**< 50, *n* = 59**	0.70 [0.21–5.22]	0.74 [0.10–2.83]	0.522	0.71 [0.21–2.52]	0.81 [0.35–1.80]	0.608	0.71 [0.21–5.22]	0.88 [0.34–2.52]	0.190
**> 50, *n* = 57**	0.68 [0.19–2.18]	0.77 [0.14–1.69]	0.694	0.65 [0.14–2.18]	0.64 [0.40–0.88]	0.852	0.78 [0.19–2.11]	0.68 [0.14–2.18]	0.894
***Male, n* = 194**	0.80 [0.08–7.61]	0.73 [0.22–5.28]	0.144	0.76 [0.33–7.61]	0.78 [0.24–5.89]	0.653	0.75 [0.21–7.61]	0.78 [0.08–5.89]	0.798
**< 50, *n* = 79**	0.75 [0.22–2.63]	0.84 [0.36–3.20]	0.683	0.74 [0.40–1.69]	0.86 [0.36–3.20]	0.499	0.74 [0.22–2.63]	0.89 [0.36–3.20]	0.537
**> 50, *n* = 115**	0.83 [0.08–7.61]	0.68 [0.22–5.28]	0.026	0.78 [0.24–5.89]	0.71 [0.24–5.89]	0.307	0.76 [0.21–7.61]	0.74 [0.08–5.89]	0.296
***ABD, n* = 39**	0.68 [0.21–2.12]	0.89 [0.33–5.28]	0.702	0.95 [0.33–5.28]	0.88 [0.53–2.12]	1.000	0.94 [0.33–5.28]	0.64 [0.21–1.33]	0.405
***No ABD, n* = 271**	0.76 [0.08–7.61]	0.74 [0.10–5.26]	0.216	0.74 [0.14–7.61]	0.73 [0.24–5.89]	0.939	0.73 [0.19–7.61]	0.78 [0.08–5.89]	0.530
***GFR > 60 mL/min, n* = 102**	0.74 [0.21–2.63]	0.70 [0.33–4.75]	0.697	0.75 [0.21–4.75]	0.63 [0.35–2.12]	0.103	0.72 [0.21–4.75]	0.78 [0.42–1.69]	0.447
***GFR < 60 mL/min, n* = 208**	0.82 [0.08–7.6]	0.77 [0.10–5.28]	0.306	0.69 [0.14–7.61]	0.82 [0.24–5.89]	0.345	0.77 [0.19–7.61]	0.78 [0.08–5.89]	0.795

ABD, adynamic bone disease; CMPF, 3−carboxy−4−methyl−5−propyl−furanpropionic acid; GFR, glomerular filtration rate; HA, hippuric acid; IAA, indole−3−acetic acid; IxS, indoxylsulfate; pCG, p−cresylglucuronide; pCS, p−cresylsulfate; r, correlation coefficient; TMAO, trimethylamine−N−oxide.

Table S12. Correlations between UT concentrations and BMD changes 12 and 24 months after transplantation, by subgroup.

	**Uremic Toxins**													
	**pCS**		**CMPF**		**IxS**		**pCG**		**HA**		**TMAO**		**IAA**	
**Subgroups**	**rho**	***p***	**rho**	***p***	**rho**	***p***	**rho**	***p***	**rho**	***p***	**rho**	***p***	**rho**	***p***
**Changes in the lumbar spine BMD (%) 12 months after transplantation**														
**All, *n* = 310**	−0.02	0.747	−0.09	0.116	+0.02	0.736	−0.03	0.624	+0.02	0.666	−0.01	0.901	−0.03	0.569
***Female, n* = 116**	−0.12	0.215	−0.05	0.595	−0.09	0.337	−0.12	0.200	+0.03	0.781	−0.01	0.891	−0.04	0.660
**< 50, *n* = 59**	−0.12	0.371	−0.01	0.943	−0.17	0.200	−0.20	.0135	−0.01	0.914	+0.11	0.399	−0.04	0.742
**> 50, *n* = 57**	−0.09	0.527	−0.18	0.197	−0.06	0.662	−0.04	0.747	+0.06	0.663	−0.20	0.149	−0.07	0.612
***Male, n* = 194**	+0.02	0.770	−0.11	0.125	+0.07	0.329	+0.01	0.870	−0.05	0.533	−0.00	0.962	−0.03	0.694
**< 50, *n* = 79**	+0.08	0.486	−0.04	0.708	+0.11	0.327	+0.11	0.337	+0.01	0.920	+0.02	0.837	+0.13	0.254
**> 50, *n* = 115**	−0.02	0.816	−0.12	0.186	+0.03	0.775	−0.05	0.613	−0.09	0.340	−0.04	0.667	−0.07	0.467
***ABD, n* = 39**	+0.27	0.104	−0.12	0.487	+0.10	0.571	+0.01	0.975	−0.16	0.354	−0.12	0.488	+0.10	0.557

No ABD, n = 271	−0.05	0.400	−0.10	0.118	+0.00	0.943	−0.03	0.602	−0.02	0.761	+0.01	0.927	−0.05	0.419
ESW, n = 41	−0.08	0.634	+0.09	0.591	+0.09	0.590	−0.01	0.947	+0.01	0.930	+0.18	0.272	+0.10	0.539
OSR, n = 269	−0.01	0.868	−0.08	0.166	+0.04	0.486	−0.02	0.787	−0.02	0.730	−0.01	0.874	−0.08	0.195
GFR > 60 mL/min, n = 102	−0.15	0.127	+0.01	0.908	−0.10	0.344	−0.02	0.831	+0.06	0.533	+0.08	0.436	−0.01	0.958
GFR < 60 mL/min, n = 208	+0.04	0.584	−0.15	0.033	+0.07	0.285	−0.02	0.744	−0.06	0.390	−0.03	0.625	−0.03	0.645
**Changes in the lumbar spine BMD (%) 24 months after transplantation**														
**All, *n* = 222**	−0.00	0.967	−0.10	0.135	−0.03	0.662	+0.03	0.621	−0.03	0.614	+0.07	0.320	−0.06	0.368
Female, n = 86	+0.02	0.857	−0.06	0.553	−0.11	0.298	+0.05	0.657	−0.02	0.869	+0.04	0.704	−0.17	0.116
**< 50, *n* = 46**	+0.00	0.980	−0.05	0.759	−0.17	0.272	+0.01	0.953	−0.14	0.371	+0.04	0.783	−0.20	0.195
**> 50, *n* = 40**	+0.06	0.715	−0.12	0.462	−0.09	0.566	+0.10	0.558	+0.10	0.526	+0.03	0.876	−0.17	0.291
Male, n = 136	−0.02	0.826	−0.12	0.148	+0.01	0.937	+0.02	0.779	−0.05	0.582	+0.08	0.361	−0.01	0.932
**< 50, *n* = 60**	+0.11	0.417	+0.00	0.997	+0.06	0.623	+0.11	0.416	+0.01	0.967	+0.12	0.354	−0.9	0.517
**> 50, *n* = 76**	−0.14	0.214	−0.17	0.135	−0.08	0.487	−0.03	0.810	−0.12	0.309	−0.01	0.964	−0.05	0.675
ABD, n = 26	+0.00	0.983	+0.30	0.134	−0.25	0.219	+0.00	0.991	−0.18	0.387	+0.00	0.997	−0.10	0.628
No ABD, n = 196	−0.00	0.960	−0.16	0.023	−0.00	0.991	+0.04	0.544	−0.03	0.724	+0.08	0.276	−0.06	0.436
ESW, n = 16	−0.19	0.475	+0.68	0.003	+0.23	0.376	+0.32	0.232	+0.36	0.171	+0.18	0.515	+0.01	0.969
OSR, n = 206	+0.02	0.798	−0.10	0.139	−0.01	0.856	+0.05	0.440	−0.03	0.697	+0.08	0.259	−0.05	0.494
GFR > 60 mL/min, n = 76	+0.04	0.751	+0.04	0.761	−0.17	0.144	+0.02	0.869	+0.01	0.925	+0.17	0.141	+0.08	0.493
GFR < 60 mL/min, n = 146	−0.02	0.797	−0.18	0.028	+0.05	0.578	+0.05	0.544	−0.06	0.501	+0.03	0.733	−0.12	0.134
**Changes in the femoral neck BMD (%) 12 months after transplantation**														
**All, *n* = 310**	−0.10	0.229	+0.10	0.218	+0.04	0.654	−0.01	0.908	−0.03	0.760	+0.07	0.397	+0.01	0.869
Female, n = 116	−0.14	0.347	+0.22	0.131	+0.23	0.124	+0.00	0.984	+0.12	0.437	+0.02	0.906	−0.10	0.485
**< 50, *n* = 59**	+0.04	0.853	+0.22	0.309	+0.18	0.417	+0.15	0.498	−0.01	0.976	+0.14	0.514	−0.11	0.599
**> 50, *n* = 57**	−0.21	0.320	−0.03	0.901	+0.13	0.560	−0.18	0.389	+0.13	0.530	−0.29	0.177	−0.21	0.330
Male, n = 194	−0.11	0.297	+0.00	0.950	+0.05	0.614	−0.03	0.766	−0.03	0.775	+0.08	0.455	+0.02	0.808
**< 50, *n* = 79**	−0.06	0.716	+0.22	0.176	−0.08	0.634	+0.00	0.994	−0.01	0.967	+0.16	0.334	+0.25	0.123
**> 50, *n* = 115**	−0.14	0.294	−0.10	0.433	−0.03	0.832	−0.05	0.701	−0.05	0.724	+0.02	0.870	−0.03	0.820
ABD, n = 39	+0.01	0.972	+0.00	0.973	−0.03	0.920	+0.06	0.806	−0.13	0.617	−0.10	0.688	−0.13	0.596
No ABD, n = 271	−0.10	0.246	+0.10	0.256	+0.05	0.569	−0.01	0.915	+0.02	0.787	+0.08	0.376	+0.04	0.686
ESW, n = 41	+0.04	0.848	+0.38	0.052	+0.31	0.136	+0.10	0.642	+0.13	0.536	+0.33	0.104	−0.07	0.730

OSR, n = 269	−0.12	0.198	+0.09	0.350	+0.01	0.907	−0.03	0.780	+0.00	0.967	+0.04	0.646	+0.03	0.747
GFR > 60 mL/min, n = 102	−0.25	0.083	+0.18	0.224	−0.05	0.711	−0.05	0.732	−0.13	0.386	+0.06	0.664	−0.04	0.779
GFR < 60 mL/min, n = 208	−0.04	0.719	+0.04	0.673	+0.10	0.357	+0.01	0.926	−0.11	0.265	+0.07	0.470	+0.05	0.658
**Changes in the femoral neck BMD (%) 24 months after transplantation**														
**All, *n* = 222**	+0.03	0.747	−0.03	0.733	+0.11	0.244	+0.06	0.533	+0.13	0.173	+0.07	0.443	+0.05	0.576
Female, n = 86	+0.03	0.859	−0.10	0.553	+0.18	0.277	+0.13	0.410	+0.33	0.038	+0.15	0.341	+0.25	0.119
**< 50, *n* = 46**	−0.12	0.594	−0.19	0.382	+0.29	0.185	−0.07	0.766	+0.26	0.238	+0.22	0.314	+0.38	0.075
**> 50, *n* = 40**	+0.22	0.399	+0.06	0.805	+0.09	0.737	+0.33	0.195	+0.37	0.145	+0.09	0.722	+0.08	0.755
Male, n = 136	+0.03	0.783	+0.02	0.893	+0.08	0.484	+0.03	0.813	+0.02	0.861	−0.01	0.946	−0.03	0.808
**< 50, *n* = 60**	+0.22	0.231	+0.37	0.041	+0.18	0.322	+0.13	0.480	+0.12	0.530	−0.01	0.975	+0.25	0.168
**> 50, *n* = 76**	−0.11	0.496	−0.05	0.772	−0.03	0.852	−0.03	0.852	−0.11	0.496	−0.03	0.856	−0.13	0.423
ABD, n = 26	+0.08	0.771	+0.02	0.942	−0.27	0.297	−0.16	0.533	−0.31	0.223	−0.00	0.998	−0.29	0.257
No ABD, n = 196	+0.03	0.798	+0.04	0.704	+0.18	0.077	+0.14	0.174	+0.19	0.069	+0.09	0.381	+0.08	0.416
ESW, n = 16	−0.24	0.458	+0.45	0.135	+0.41	0.187	+0.25	0.435	+0.39	0.209	+0.79	0.002	+0.33	0.288
OSR, n = 206	+0.06	0.531	+0.05	0.630	+0.09	0.356	+0.06	0.580	+0.11	0.267	+0.03	0.759	+0.04	0.669
GFR > 60 mL/min, n = 76	+0.21	0.182	+0.09	0.552	+0.14	0.372	−0.07	0.642	+0.02	0.897	+0.00	0.989	+0.07	0.650
GFR < 60 mL/min, n = 146	−0.05	0.662	−0.13	0.280	+0.11	0.383	+0.15	0.218	+0.21	0.090	+0.11	0.375	+0.04	0.727
**Changes in the total hip BMD (%) 12 months after transplantation**														
**All, *n* = 310**	−0.05	0.389	−0.02	0.770	−0.02	0.763	−0.04	0.545	−0.07	0.257	−0.00	0.976	+0.07	0.238
Female, n = 116	+0.04	0.686	+0.06	0.547	−0.17	0.094	−0.05	0.641	+0.04	0.712	−0.02	0.834	+0.11	0.295
**< 50, *n* = 59**	+0.07	0.618	−0.03	0.833	+0.00	0.986	−0.22	0.123	−0.05	0.731	+0.28	0.052	+0.09	0.540
**> 50, *n* = 57**	+0.09	0.538	+0.05	0.727	+0.28	0.048	+0.09	0.557	+0.08	0.594	−0.31	0.025	+0.08	0.575
Male, n = 194	−0.08	0.286	−0.03	0.653	−0.07	0.333	−0.03	0.702	−0.10	0.176	+0.02	0.792	+0.08	0.311
**< 50, *n* = 79**	−0.14	0.260	−0.09	0.479	−0.14	0.235	−0.13	0.275	−0.21	0.078	+0.00	0.968	−0.03	0.778
**> 50, *n* = 115**	−0.03	0.764	−0.01	0.956	−0.02	0.869	+0.05	0.587	+0.01	0.915	+0.04	0.706	+0.13	0.182
ABD, n = 39	−0.01	0.951	+0.04	0.832	−0.34	0.064	+0.01	0.953	−0.35	0.051	−0.11	0.552	−0.12	0.531
No ABD, n = 271	−0.05	0.396	−0.04	0.506	+0.02	0.792	−0.05	0.452	−0.06	0.321	+0.01	0.893	+0.10	0.112
ESW, n = 41	+0.13	0.426	+0.41	0.012	+0.27	0.109	+0.09	0.613	+0.17	0.323	−0.06	0.716	+0.19	0.250
OSR, n = 269	−0.09	0.186	−0.06	0.362	−0.05	0.436	−0.05	0.432	−0.11	0.098	+0.01	0.930	+0.04	0.572

GFR > 60 mL/min, n = 102	−0.17	0.117	+0.03	0.802	−0.17	0.105	−0.19	0.078	−0.07	0.500	+0.20	0.058	−0.09	0.398
GFR < 60 mL/min, n = 208	+0.01	0.935	−0.04	0.570	+0.08	0.302	+0.03	0.673	−0.07	0.367	−0.09	0.210	+0.13	0.076
**Changes in the total hip BMD (%) 24 months after transplantation**														
**All, *n* = 222**	−0.05	0.509	−0.02	0.730	+0.04	0.570	+0.01	0.868	+0.02	0.743	+0.03	0.671	−0.01	0.944
Female, n = 86	−0.02	0.886	−0.06	0.610	+0.06	0.598	−0.07	0.561	+0.12	0.303	+0.05	0.690	+0.09	0.437
**< 50, *n* = 46**	+0.06	0.736	+0.08	0.618	−0.07	0.687	−0.25	0.132	+0.13	0.403	+0.23	0.174	+0.08	0.639
**> 50, *n* = 40**	−0.04	0.834	−0.24	0.167	+0.22	0.206	+0.14	0.435	+0.10	0.562	−0.21	0.225	+0.08	0.628
Male, n = 136	−0.05	0.596	+0.01	0.938	+0.06	0.519	+0.05	0.551	+0.02	0.984	+0.03	0.733	−0.05	0.592
**< 50, *n* = 60**	−0.05	0.704	−0.12	0.376	+0.04	0.755	−0.03	0.802	−0.06	0.652	−0.05	0.704	−0.17	0.203
**> 50, *n* = 76**	−0.05	0.699	+0.11	0.362	+0.04	0.735	+0.11	0.348	+0.06	0.637	+0.11	0.380	−0.00	0.996
ABD, n = 26	+0.11	0.633	+0.25	0.276	−0.26	0.245	+0.06	0.794	−0.17	0.460	+0.02	0.926	−0.17	0.474
No ABD, n = 196	−0.07	0.378	−0.06	0.435	+0.08	0.299	+0.00	0.975	+0.03	0.679	+0.03	0.692	+0.01	0.857
ESW, n = 16	−0.13	0.625	+0.48	0.058	+0.47	0.069	+0.32	0.221	+0.40	0.124	+0.30	0.260	+0.34	0.196
OSR, n = 206	−0.04	0.613	−0.03	0.688	+0.04	0.612	+0.02	0.839	+0.02	0.787	+0.03	0.718	−0.01	0.919
GFR > 60 mL/min, n = 76	−0.02	0.871	−0.04	0.739	−0.01	0.942	−0.00	0.995	+0.06	0.635	+0.12	0.332	−0.12	0.364
GFR < 60 mL/min, n = 146	−0.06	0.485	−0.05	0.575	+0.06	0.479	+0.03	0.694	−0.00	0.994	−0.01	0.888	+0.05	0.530

ABD, adynamic bone disease; CMPF, 3-carboxy-4-methyl-5-propyl-furanpropionic acid; ESW, early steroids withdrawal; GFR, glomerular filtration rate; HA, hippuric acid; IAA, indole-3-acetic acid; IxS, indoxylsulfate; OSR, other steroid regimens; pCG, p-cresylglucuronide; pCS, p-cresylsulfate; r, correlation coefficient; TMAO, trimethylamine-N-oxide.
